# Effects of biochar and arbuscular mycorrhizal fungi on winter wheat growth and soil N_2_O emissions in different phosphorus environments

**DOI:** 10.3389/fpls.2022.1069627

**Published:** 2022-12-14

**Authors:** Zhuo Hao, Zhijie Dong, Shuo Han, Aiping Zhang

**Affiliations:** Institute of Environment and Sustainable Development in Agriculture, Chinese Academy of Agricultural Sciences, Beijing, China

**Keywords:** denitrification, root morphology, foraging strategy, nitrogen retention, plant-soil-microbe interaction, leaf Mn concentration

## Abstract

**Introduction:**

Promoting crop growth and regulating denitrification process are two main ways to reduce soil N_2_O emissions in agricultural systems. However, how biochar and arbuscular mycorrhizal fungi (AMF) can regulate crop growth and denitrification in soils with different phosphorus (P) supplies to influence N_2_O emission remains largely unknown.

**Method:**

Here, an eight-week greenhouse and one-year field experiments biochar and/or AMF (only in greenhouse experiment) additions under low and high P environments were conducted to characterize the effects on wheat (Triticum aestivum L.) growth and N_2_O emission.

**Results:**

With low P supply, AMF addition decreased leaf Mn concentration (indicates carboxylate-releasing P-acquisition strategies), whereas biochar addition increased leaf Mn concentration, suggesting biochar and AMF addition regulated root morphological and physiological traits to capture P. Compared with low P supply, the high P significantly promoted wheat growth (by 16-34%), nutrient content (by 33-218%) and yield (by 33-41%), but suppressed soil N_2_O emissions (by 32-95%). Biochar and/or AMF addition exhibited either no or negative effects on wheat biomass and nutrient content in greenhouse, and biochar addition promoted wheat yield only under high P environment in field. However, biochar and/or AMF addition decreased soil N_2_O emissions by 24-93% and 32% in greenhouse and field experiments, respectively. This decrease was associated mainly with the diminished abundance of N_2_O-producing denitrifiers (nirK and nirS types, by 17-59%, respectively) and the increased abundance of N_2_O-consuming denitrifiers (nosZ type, by 35-65%), and also with the increased wheat nutrient content, yield and leaf Mn concentration.

**Discussion:**

These findings suggest that strengthening the plant-soil-microbe interactions can mitigate soil N_2_O emissions via manipulating plant nutrient acquisition and soil denitrification.

## 1 Introduction

As a significant greenhouse gas, nitrous oxide (N_2_O) has the global warming potential 298 times higher than the equivalent mass of CO_2_ in the atmosphere (Philibert et al., 2013). Due to high rates of organic and inorganic nitrogen (N) fertilization in agricultural production, about 60% of global anthropogenic N_2_O emissions is derived from agricultural soils (Davidson, 2009). Among the three main processes (denitrification, nitrification and nitrifier-denitrification) that produce N_2_O in soils (Kool et al., 2011), denitrification is the primary source (Case et al., 2015). Generally, there are two major ways to decrease N_2_O emissions in agriculture (Jiang et al., 2020): (i) promote the crop growth and N uptake, thus reducing availability of N for N_2_O production, and (ii) alter the N cycling processes (e.g. denitrification) *via* regulating the abundance and activity of relevant microorganisms. Thus, linking plant nutrient-acquisition strategy and soil microbial community (composition and activity) can improve our understanding of mechanisms effective in suppressing N_2_O emissions under different management practices.

The denitrification rate and its contribution to N_2_O emissions are influenced strongly by soil conditions (e.g. soil pH, soil water content, availability of oxygen, C, N, P, etc.). The N_2_O:N_2_ product ratio in denitrification was correlated negatively with soil pH, labile C availability, and soil water-filled pore space, and positively with soil NO_3_^-^ content (Senbayram et al., 2012). As a soil amendment, biochar application can modify these soil conditions. Biochar, being C-rich and porous, can improve soil aeration, reduce soil bulk density, enhance soil water holding capacity, and adsorb nutrients (such as P) from soil to change C, N and P availability (Zhang et al., 2016; Jiang et al., 2020; Sun et al., 2022). Biochar application can also increase pH of acid soils, but has no effect or can decrease pH of alkaline soils (Lehmann and Joseph, 2009; Dong et al., 2022). In addition, biochar can promote crop root activity and shoot growth, thus enhancing plant nutrient absorption and decreasing N content in soil (Liu et al., 2021; An et al., 2022). Thus, biochar application in agriculture is considered an efficient strategy to reduce N_2_O emissions *via* modulating denitrification and crop growth.

Different P conditions can influence soil N pools and cycling processes *via* regulating plant growth and microbial activity (Xiao et al., 2022a; Xiao et al., 2022b). In P-limited environments, P addition enhanced soil N immobilization and retained more N in the plant-soil system by promoting plant and microbial growth, thus suppressing N_2_O emission (Shen and Zhu, 2022; Wang et al., 2022). In soils with different P supplies, plants can adjust P-acquisition strategies by regulating root morphology (e.g. root diameter, specific root length) and the amount and composition of root exudates (Wang et al., 2021), which may influence the soil microbial community composition and activity and thus N_2_O emission (Coskun et al., 2017; Abalos et al., 2019). For instance, P enrichment may stimulate the activity of denitrifiers and nitrifiers (Ullah et al., 2016) or alter microbial community composition regarding the taxa involved in N_2_O production (Liu et al., 2012). In addition, P enrichment can increase water consumption by plants, which may lead to decreased soil moisture and diminished N_2_O emission (Chen et al., 2017). Thus, linking the plant nutrient acquistion stategy and soil functional microbiome may advance our understanding of the effects of different P supplies on plant growth and soil N_2_O emissions.

Arbuscular mycorrhizal fungi (AMF) benefit their host plant regarding nutrient uptake (primary P, N and Zn) from soil (Smith and Read, 2008). AMF inoculation can increase microbial biomass N and plant biomass, thus reducing the availability of N substrates (NH_4_^+^ and NO_3_^-^) in soil for N_2_O producers, thus decreasing N_2_O emissions (Bender et al., 2014; Storer et al., 2018; Shen and Zhu, 2021). AMF may also influence soil aggregation (Morris et al., 2019) and soil water relations (Augé, 2004), and increase oxygen diffusion towards the interiors of soil aggregates, thus affecting denitrification (Okiobe et al., 2022). To date, studies have showed that AMF can decrease the abundance of the *nirK* type denitrifiers (that produce N_2_O) and increase the abundance of *nosZ* type (that consume N_2_O), thus hampering denitrification (Bender et al., 2014; Gui et al., 2021; Zhao et al., 2021).

Based on the current knowledge, a reasonable hypothesis is that biochar, P and AMF may interact to suppress N_2_O production by modifying the plant growth and abundance of microorganisms associated with the N cycle. Plants usually increase root/shoot ratio, specific root length and carboxylate exudation and decrease root tissue density to enhance their capacity to acquire P in P-limited environments (Lambers et al., 2006; Shen et al., 2011). However, the large amounts of carboxylate exudation may stimulate the growth of microorganisms, including the functional microorganisms involved in denitrification (Coskun et al., 2017; Abalos et al., 2019). In the P-limited environments, AMF inoculation can reduce exudation from their host roots but improve host plant nutrient acquisition, whereas in P-rich environments AMF inoculation may inhibit plant growth (Smith et al., 2011; Ryan et al., 2012; Johnson et al., 2015; Zhang et al., 2021). On the other hand, biochar can adsorb P to decrease P availability in soil in both low- and high-P environments (Dai et al., 2016). Hence, biochar application may govern the AMF effects on plant growth *via* changing the P availability. Earlier studies have also shown that biochar can promote the AMF symbiosis *via* changing soil nutrient availability, altering the activity of specific microorganisms that have effects on mycorrhizae, and serving as a refuge for fungi to colonizing plants (Warnock et al., 2007; Lehmann et al., 2011). To date, how the interaction among biochar, P and AMF modifies plant growth and denitrification in soil, and thus influences N_2_O emission, remains obscure.

Wheat is one of the most widely cultivated crops in the world and is highly sensitive to soil P availability (Li et al., 2014), with AMF having negative effects on wheat growth at the high P supply (Efthymiou et al., 2018; Ryan and Graham, 2018). In the present study, we set up a fully factorial greenhouse experiment and a field experiment. Wheat was grown with or without biochar and/or AMF at the low and high P supply. The study was aimed at testing the following hypotheses: (i) high P supply can enhance plant growth and N uptake and reduce soil N_2_O emissions; (ii) AMF may increase crop growth and inhibit key denitrification microorganisms to decrease soil N_2_O emissions at the low P supply, whereas at the high P supply, AMF may inhibit wheat growth and diminish the mitigating effect on N_2_O emission; and (iii) biochar can suppress N_2_O emission *via* directly inhibiting the key denitrification genes, and *via* directly influence wheat growth or indirectly modifying the effect of AMF on wheat growth at differential P supply.

## 2 Materials and methods

### 2.1 Experimental treatments

#### 2.1.1 Greenhouse experiment

A calcareous loamy soil (0-20 cm) was collected from Shangzhuang experimental station (40°08′ N, 116°10′ E) of China Agricultural University. The soil contained 17.8 g kg^-1^ organic matter, 870 mg kg^-1^ N, 2.9 mg kg^-1^ Olsen P, 156 mg kg^-1^ available K, and had pH value 7.8 (soil:water ratio was 1:5). The soil was air dried, sieved (2 mm) and sterilized by radiation with ^60^Co γ-rays at 10 kGy to eliminate indigenous AMF.

A greenhouse experiment was conducted with two phosphorus rates (40 and 300 mg P kg^-1^ soil as KH_2_PO_4_, denoted hereafter as P40 and P300, respectively), two mycorrhizal (AMF) treatments (with and without inoculation with *Rhizophagus irregularis*) and two biochar (BC) rates (with and without maize straw BC). There were five replicates per treatment. To achieve the same soil K addition in the two P treatments, K_2_SO_4_ was supplied at 327 mg K kg^-1^ soil in the P40 treatment. Furthermore, the mineral nutrients were added to all treatments (per kg soil) as follows: 200 mg of N (as KNO_3_), 50 mg of Mg (as MgSO_4_), 5 mg of Zn (as ZnSO_4_), and 2 mg of Cu (as CuSO_4_). The nutrients were uniformly mixed in soil before placing the soil in plastic pots (15 cm in diameter, 15 cm in height; 2 kg of soil pot^-1^). The glasshouse temperature range was 15–20°C with 10–12 h daylight throughout the wheat growth period.

Biochar was derived from maize straw residue at 450°C, and was provided by Mingchen Sanitation Equipment Co. LTD, Shandong Province, China. Biochar contained (per kg) 9.48 g total N, 1.34 g total P and 530 g carbon; pH was 9.7. Biochar was added at 3.5 g kg^-1^ soil (equivalent to 9 t ha^-1^) and was mixed thoroughly.

The AMF *Rhizophagus irregularis* was propagated on wheat plants growing in a 1:5 mixture (w/w) of river sand and zeolite in a greenhouse for 4 months. The inoculum used in the present study included substrate containing spores (150 g^-1^ potting substrate), mycelium and fine-root segments. The inoculum was added at 20 g kg^-1^ soil in the AMF treatment. The 10 mL of microbial wash filtrate from unsterilized soil was added to all pots to minimize differences in microbial communities among treatments (Martínez-García et al., 2017).

Wheat seeds (JiMai 22) were surface sterilized by stirring in 10% (v/v) hydrogen peroxide for 10 min and in 70% (v/v) ethanol for 3 min, followed by rinsing at least five times in deionized water. Six uniformly germinated seeds were sown into each pot and were thinned after emergence to four seedlings similar in size. The pots were watered daily and weighed every 2 days to adjust soil moisture to 18-20% (w/w).

#### 2.1.2 Field experiment

A one-year field experiment with four-treatment was designed to test the effects of biochar and P addition mitigate N_2_O emissions. The study site is located in the Shunyi District, Beijing. The soil contained 9.68 g kg^-1^ organic matter,1.32 g kg^-1^ N, 6.48 mg kg^-1^ Olsen P, 73.6 mg kg^-1^ available K in 0-20 cm soil depth and a pH of 8.05. The field plots were conducted with a completely randomized block design with four treatments: BC0_P0 (0 t hm^-2^ biochar, 0 kg hm^-2^ P), BC0_P+ (52 kg hm^-2^ P), BC+_P0 (9 t hm^-2^ biochar), BC+_P+ (9 t hm^-2^ biochar, 52 kg.hm^-2^ P). Before sowing of winter wheat in 2020, the amount of biochar and P in four treatments were supplied every year. Sixteen plots were arranged with four replicates, and every plot size was 180 m^2^ (6 m × 30 m).

In field experiment, wheat (JiMai 22) was sown on 12 October 2020 and harvested on 6 June 2021. The P fertilizer input amount of each treatment was 0 (P0) and 52 kg P hm^-2^ (P+) (Ca(H_2_PO_4_)_2_), K 124 kg.hm^-2^ (K_2_SO_4_), N 112 kg hm^-2^ (KNO_3_), respectively. N fertilizer was uniformly applied at a 1:1 ratio according to topdressing (15 March 2021). Each treatment was irrigated after fertilization.

### 2.2 Gas collection and measurement

#### 2.2.1 Greenhouse experiment

Seven weeks after sowing, nutrients solution with 200 mg kg^-1^ KNO_3_ was added in each pot, and the soil moisture adjusted to 30% (w/w), which was beneficial to denitrification process but had little effect on wheat growth during a week of sampling N_2_O. After 24 hours, N_2_O samples were collected from each pot by using a PVC cylindrical chamber (diameter of 16 cm and height of 50 cm), with a base having a 5 cm groove to which water was added to seal the system during sample collection. The gas in the chamber was sampled starting at 9:00 and then again starting at 15:00 each day for a week; during this period, the soil moisture was kept at 30% (w/w) by adding water. The four 50 mL gas samples were extracted by plastic syringes at 0, 10, 20 and 30 min in each of the two daily sampling periods.

#### 2.2.2 Field experiment

N_2_O samples were collected by using a PVC box chamber (50cm×50cm×50cm) with a thickness of 1.5 mm and a base (50 cm × 50 cm × 15 cm). The gas was collected twice a week after fertilization, irrigation, rain and snow before the booting stage. Afterwards, the collection frequency decreased to once every 15 days during the fallow period. Gas samples were taken between 09:00 to 11:00 am. During this period, the four 200 mL gas samples were extracted by plastic syringes at 0, 10, 20 and 30 min for each plot.

#### 2.2.3 N_2_O measurement

The measurement of N_2_O was done by gas chromatography (Agilent 7890A; Agilent, USA) with an electron capture detector (ECD) at 350°C (Liu et al., 2020). The hourly fluxes (F, μg m^-2^ h^-1^) of N_2_O were calculated according to Shi et al. (2019):

F= ρ×(V/A)×(ΔC/Δt)×(273/(273+T))

where ρ is the density of N_2_O under standard conditions, V is the chamber volume (m^3^), A is the area covered by chamber (m^2^), ΔC/Δt is the change in N_2_O concentration in the chamber over time, and T is the chamber air temperature.

The cumulative N_2_O emission (E, mg m^-2^) was calculated as follows:

E=(F_i_+F_i+1_)/2 ×24×10^-3^×T

where i is the i^th^ measurement, 24 × 10^-3^ was used for unit conversion, and T was the number of days of sample collection.

The *nosZ/(nirK+nirS)* was the *nosZ* and *(nirK+nirS)* gene copy numbers raito. *nosZ* associated with N_2_O consumption in the process of denitrification, and *(nirK+nirS)* was significantly positively correlated with the denitrification rates (Xuan et al., 2022). The ratio is used to characterize the degree of N_2_O emission inhibition (Liu et al., 2020)

### 2.3 Plant harvest and soil sampling

#### 2.3.1 Greenhouse experiment

Eight weeks after sowing (Feekes 3, tillering stage), shoots were cut at the soil surface, and roots were extracted from the soil. Shoots were rinsed in distilled water and then oven-dried at 75°C for 72 h, weighed and ground to fine powder. Nitrogen and phosphorus concentrations in shoots were determined after digestion with a mixture of 5 mL of concentrated H_2_SO_4_ and 8 mL of 30% v/v H_2_O_2_. Shoot N was analyzed by the Kjeldahl method and P by the molybdovanadophosphate method. Leaf Mn concentration was used to indicate carboxylate-releasing P-acquisition strategies (Lambers et al., 2015) and was determined directly by Atomic Absorption Spectroscopy (AAS, GBC 904 AvantaVer 1.33, Australia).

Roots were washed under running water and were scanned at the resolution of 400 dpi (EPSON 1680) (Epson, Long Beach, CA, USA). Root images were analyzed using a WinRHIZO image system (WinRHIZOPro2004b) (Null, Regent, Canada) to calculate root length and diameter (Wang et al., 2020). After scanning, a weighed subsample of the root system was cleared and stained with trypan blue to determine mycorrhizal colonization by the method of Kormanik and McGraw (1982). The roots were dried at 75°C for 72 h and weighed. Calculations of root tissue density and specific root length were done according to Li et al. (2013).

The soil samples were collected on the same day as the gas samples. The soil was sieved (2 mm) and separated into two subsamples: one was kept at -80°C for quantitative PCR assay and the other one was stored at 4°C for determining C, P and N content. Soil NO_3_^-^-N and NH_4_^+^-N were extracted with 0.01 mol L^-1^ CaCl_2_ solution and determined by a AA3 flow analyzer (Braun and Lubbe, Norderstedt, Germany). Soil available P was measured by the Olsen method. Soil organic carbon was analyzed by wet digestion with 10% w/w H_3_PO_4._

In order to test the effects of P, BC and AMF on denitrification, the copy numbers of the key genes of copper nitrite reductases (*nirS*, *nirK*) and nitrous oxide reductase (*nosZ*) were determined. DNA was extracted using a Fast DNA Spin Kit for Soils (MP Biomedicals, Solon, OH, USA) from 0.5 g of soil. The quantitative polymerase chain reaction (q-PCR) amplification primers for *nirK*, *nirS* and *nosZ* genes and the reaction conditions are shown in **Table S1**. The qPCR was performed using a CFX96 Optical Real-Time Detection System (Bio-Rad Laboratories, Hercules, CA, USA).

#### 2.3.2 Field experiment

At wheat maturity, wheat yield was measured in each treatment plot. Soil samples (0–20 cm) were collected when soil N_2_O emissions tended to be stable after fertilization. Five soils from two diagonal lines through each plot were collected and pooled into one composite sample. The soil was sieved (2 mm) and was kept at -80°C, and then used to measure the functional genes of denitrification.

### 2.4 Statistical analyses

Two-way ANOVA was performed to test for effects of AMF and biochar addition on N_2_O emission, gene abundance, root traits and soil properties (*P* ≤0.05) at low or high P supply. *Post-hoc* Tukey HSD tests were performed to determine significant differences. If the two-way interaction AMF× BC was significant, the complete data was shown; if not, only the significant main effects were shown. Significant differences between the AMF and no AMF inoculation at each P supply or between BC and no BC addition at each P supply were based on the t-test (*P* ≤0.05). Pearson’s correlation analysis was used to test the relationships among the N_2_O emission, abundance of genes, root traits and soil properties. The random forest model in R studio software was used to analyze the comprehensive effects of genes, root traits and soil properties on the soil N_2_O emissions.

Two-way ANOVA was performed to test for effects of P and biochar addition on yield, N_2_O emission and gene abundance (*P* ≤0.05) in the field experiment. *Post-hoc* Tukey HSD tests were performed to determine significant differences. Pearson’s correlation analysis was used to test the relationships among the yield, N_2_O emission and gene abundance.

## 3 Results

### 3.1 Shoot growth properties

**Figure 1:**
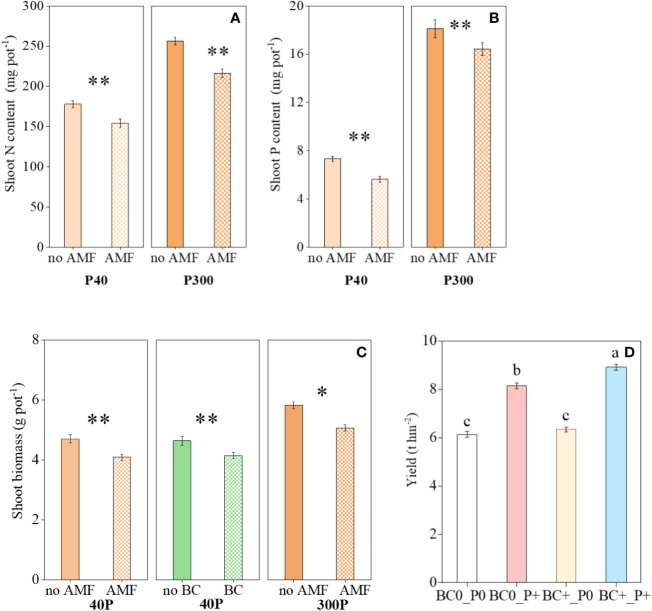
Variation in the **(A)** shoot N content, **(B)** shoot P content and **(C)** shoot biomass as influenced by P rates (40 and 300 mg kg^−1^ soil) and AMF and BC additions in the greenhouse experiment. Each value is the mean of five replicates (±SE). **(D)** Variation in wheat yield in field experiment. Each value is the mean of four replicates (±SE). *P ≤ 0.05 and **P < 0.01.

The interaction AMF × BC was not significant for shoot biomass and shoot N and P contents (**Table 1**). The shoot biomass was influenced significantly by mycorrhiza at both P rates, but the biochar main effect was a significant source of variation for shoot biomass at P300 only (**Table 1** and **Figure 1**). The mycorrhizal main effect was a significant source of variation for shoot N content and shoot P content at both P40 and P300 (**Table 1** and **Figure 1**). In the presence of mycorrhiza, shoot biomass and shoot N content were significantly lower than in the non-mycorrhizal treatment regardless of the P rate. By contrast, AMF addition was associated with a decrease in shoot P content at P40 and an increase at P300 (**Figure 1**). Compared to the low P supply, the high P significantly promoted shoot growth (by 16-34%), shoot N content (by 33-47%) and shoot P content (by 145-218%) (calculated from **Figure 1**). In the field experiment, the interaction BC×P was significant for wheat yield (**Table 2**). Compare to no P addition treatment, wheat yield increased significantly (by 33% and 41%, respectively) in P addition treatments.

**Table 1:** The P values from 2-way ANOVA (AMF=arbuscular mycorrhizal inoculum and BC=biochar) at the two P rates (P40 and P300) regarding soil N_2_O emissions, gene abundance, and plant and soil properties in the greenhouse experiment.

P rates		P40			P300		
Variables		AMF	BC	AMF×BC	AMF	BC	AMF×BC
N_2_O		<0.001	<0.001	<0.001	0.085	0.023	0.265
*nirK*		0.56	0.234	0.539	0.298	<0.001	0.002
*nirS*		<0.001	<0.001	0.72	0.517	0.003	0.119
*nosZ*		<0.001	<0.001	<0.001	0.007	<0.001	0.881
*nosZ/(nirK+nirS)*		<0.001	0.004	<0.001	0.184	<0.001	0.031
TRL		0.375	<0.001	0.028	<0.001	<0.001	0.823
RD		<0.001	<0.001	<0.001	0.026	0.003	<0.001
SRL		0.053	0.948	0.059	<0.001	<0.001	0.065
RTD		0.001	0.003	0.013	<0.001	0.003	0.013
RB		0.101	<0.001	0.009	0.251	0.001	0.039
SB		<0.001	0.001	0.254	<0.001	0.216	0.122
MC%		<0.001	0.001	0.001	<0.001	0.013	0.013
Shoot P content		<0.001	0.115	0.434	0.022	0.238	0.906
Shoot N content		0.004	0.593	0.699	<0.001	0.127	0.065
Mn		<0.001	0.032	0.263	0.001	0.634	0.002
Olsen P		0.032	<0.001	0.001	0.056	<0.001	0.002
NO_3_^-^-N		0.009	0.827	0.015	<0.001	0.968	0.432
NH_4^+^-_N		0.003	<0.001	0.844	0.006	<0.001	0.129
STN		0.031	0.6	0.674	0.083	0.32	0.483
SOC		0.046	<0.001	0.63	0.011	<0.001	0.454

TRL, total root length; RV, root volume; MC%, mycorrhizal colonization; RD, root diameter; SRL, specific root length; RTD, root tissue density; RB, root biomass; R/S, root/shoot ratio; SB, shoot biomass; Mn, mature leaf Mn concentrations; N/P, shoot N content/shoot P content ratio; STN, soil total nitrogen; SOC, soil organic carbon.

**Table 2:** Effects of P and biochar application on N_2_O emission, *nirK, nirS* and *nosZ* gene copy numbers and *nosZ/(nirK+nirS)* in field experiment.

Treatment	*n*	*nirK*	*nirS*	*nosZ*	*nosZ/(nirK+nirS)*
BC0_P0	4			(6.25 ± 0.5)×10^7^b	0.06 ± 0.01b
BC0_P+	4			(6.5 ± 0.27)×10^7^b	0.09 ± 0.01b
BC+_ P0	4			(7.1 ± 0.34)×10^7^b	0.10 ± 0.01b
BC+_ P+	4			(9.0 ± 0.23)×10^7^a	0.19 ± 0.03a
BC0	8	(3.65 ± 0.44)×10^8^	(5.51 ± 0.25)×10^8^		
BC+	8	(2.82 ± 0.34)×10^8^	(3.56 ± 0.30)×10^8^		
P0	8	(4.18 ± 0.26)×10^8^	(4.97 ± 0.40)×10^8^		
P+	8	(2.28 ± 0.16)×10^8^	(4.10 ± 0.46)×10^8^		
***p* value**					
BC		<0.001	<0.001	0.001	0.002
P		<0.001	0.02	0.02	0.004
BC×P		0.16	0.57	0.041	0.049

Lowercase letter in the same column and in the same growth season represents significant differences among experimental treatments at the level of 0.05 based on two-way ANOVA. Where the BC×P interaction was nonsignificant, only the main effects were presented.

### 3.2 Root morphological and physiological traits

**Figure 2:**
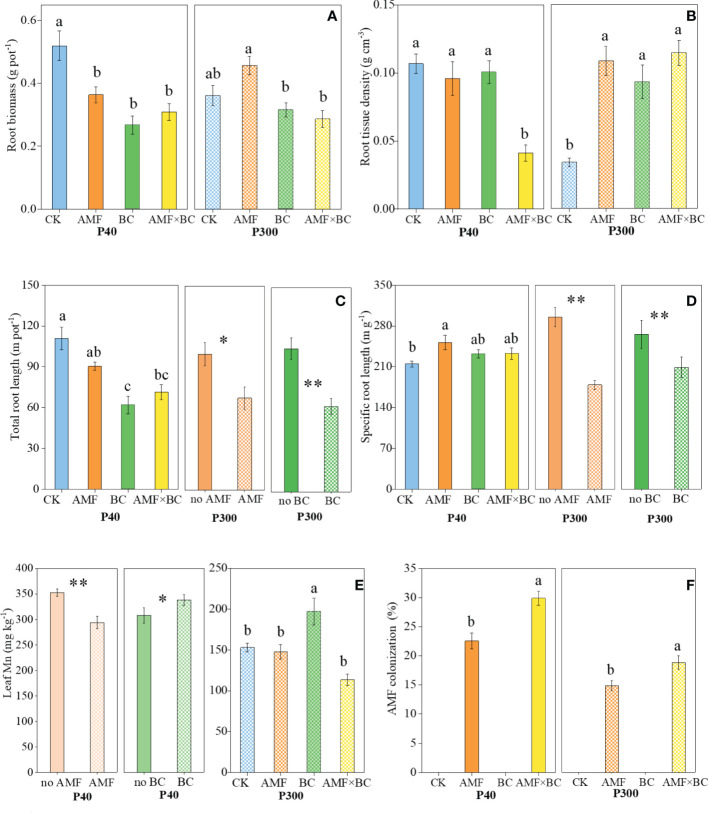
Variation in the root biomass and morphological traits **(A)** root biomass, **(B)** root tissue density, **(C)** total root length, **(D)** specific root length, **(E)** leaf Mn concentrations, and **(F)** AMF colonization as influenced by P rates (40 and 300 mg kg^−1^ soil) and AMF and BC additions. Each value is the mean of five replicates (±SE). For a given P rate, different letters in each graph denote significant difference among treatments (*P* ≤ 0.05). *P ≤ 0.05 and **P < 0.01.

Root biomass and root tissue density were significantly influenced by the interaction AMF×BC at P40 and P300 (**Table 1**). At the low P supply, compared to the control, root biomass decreased significantly (by 30% to 48%) in the other treatments (**Figures 2A, B**). Regarding root tissue density, contrasting results were obtained at two P supplies. Compared to the other treatments, root tissue density was more than 2-fold lower in the combined AMF and BC treatment at P40 and in the control at P300 (**Figure 2B**). Root tissue density had a significantly negative correlation with root diameter in both P40 and P300 (**Table S2**).

The AMF×BC interaction was a significant source of variation for total root length and specific root length at P40 only (**Table 1**). In the combined AMF and BC treatment, total root length was 36% lower, and specific root length was 8.4% higher, than the control at P40 (**Figure 2C**). At the high P supply, both main effects significantly influenced total root length and specific root length (**Table 1**). The total root length and specific root length decreased significantly (by 32% and 65%, respectively) with mycorrhizal inoculation. Biochar addition decreased total root length by 40% and specific root length by 22% (**Figures 2C, D**).

Leaf Mn concentration was significantly influenced by the interaction AMF×BC at P300 only (**Table 1**). Compared to the control, Mn concentration was decreased significantly in the combined AMF and BC treatment at P300 (**Figure 2E**). At the low P supply, both main effects significantly influenced Mn concentration (**Figure 2E**) that increased by 10% with biochar addition and decreased by 17% with mycorrhizal inoculation.

### 3.3 AMF colonization

The mycorrhizal colonization was significantly influenced by the AMF × BC interaction (**Table 1**). No root colonization was detected in the treatments without AMF addition. AMF colonization tended to be higher at P40 than P300. In the given P treatment (**Figure 2F**) biochar addition increased AMF colonization by 27-33% compared to the AMF treatment (**Figure 2F**).

### 3.4 Soil N_2_O emissions

**Figure 3:**
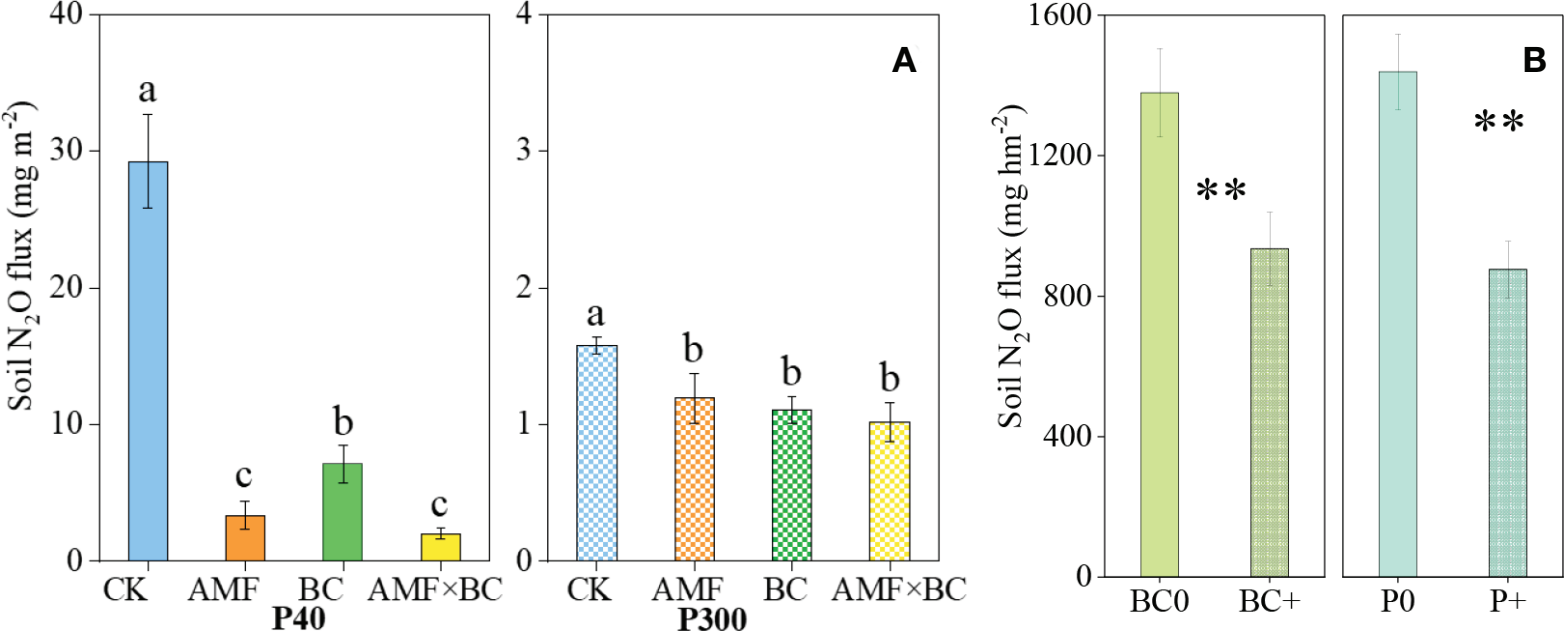
**(A)** Variation in the cumulative emissions of N2O as influenced by P rates (40 and 300 mg kg^−1^ soil) and AMF and BC additions in the greenhouse experiment. Each value is the mean of five replicates (±SE). For a given P rate, different letters in each graph denote significant difference among treatments. **(B)** Variation in the cumulative emissions of N_2_O as influenced by P and BC additions in field experiment. Each value is the mean of four replicates (±SE). **P < 0.01.

The soil N_2_O emissions was significantly influenced by the AMF×BC interaction at P40 only (**Table 1**). At the low P supply, compared to the control, the soil N_2_O emissions decreased significantly (by 88%, 76% and 93%) in, respectively, the AMF, BC and the combined AMF and BC treatments (**Figure 3A**).

The biochar main effect was a significant source of variation for soil N_2_O emissions at P300. Compared to control, the soil N_2_O emissions decreased significantly (by 30% and 24%, respectively) with biochar addition and mycorrhizal inoculation. Compared to the low P supply, the high P significantly suppressed soil N_2_O emissions by 50-95% (calculated from **Figure 3A**).

In the field experiment, the soil N_2_O emissions was significantly influenced by P and biochar addition (**Table 2**). Compared to no biochar and no P environments, biochar and P addition reduced soil N_2_O emissions by 32% and 39%, respectively (**Figure 3B**).

### 3.5 Soil properties

The AMF×BC interaction was a significant source of variation for soil NO_3_^-^-N at P40 only (**Table 1**). The mycorrhizal main effect was a significant source of variation for soil NO_3_^-^-N at P300. At the low P supply, the soil NO_3_^–^N was 48% higher with mycorrhizal inoculation than the control, but decreased by 27% in the presence of mycorrhiza compared to the non-mycorrhizal treatment at P300 (**Figure S1A**).

The interaction AMF×BC interaction was not significant for the soil NH_4^+^-_N (**Table 1**). The soil NH_4_^+^-N was significantly influenced by mycorrhiza and biochar addition at both P rates. Biochar addition was associated with an increase in the soil NH_4^+^-_N at P40 and P300. Mycorrhizal inoculation was associated an increase at P40 and a decrease at P300 (**Figure S1B**).

### 3.6 Abundance of nitrification and denitrification genes in the soil

**Figure 4:**
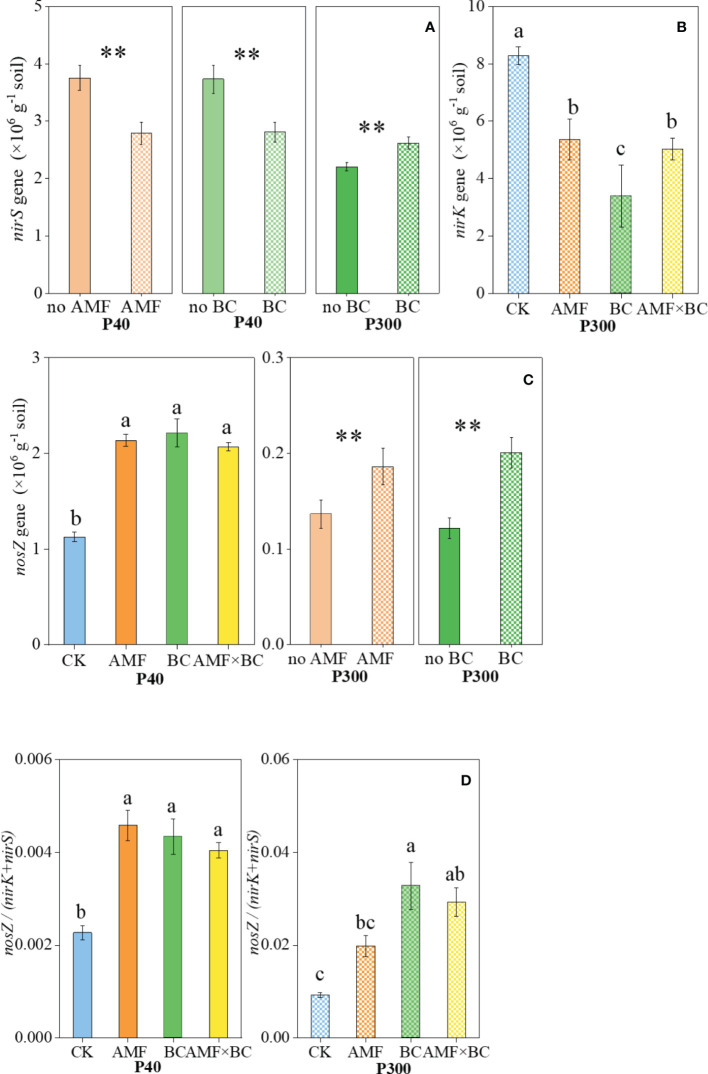
Variation in the **(A)** nirS, **(B)** nirK, and **(C)** nosZ gene copy numbers and **(D)** the ratio of nosZ/(nirK+nirS) as influenced by P rates (40 and 300 mg kg^−1^ soil) and AMF and BC additions. Each value is the mean of five replicates (±SE). For a given P rate, different letters in each graph denote significant difference among treatments (P ≤ 0.05). **P < 0.01. Note an order of magnitude difference in the scale on the Y-axis.

The abundance of *nirS* gene was significantly influenced by mycorrhizal addition at P40 only, but the biochar main effect was a significant source of variation for *nirS* gene abundance at both P rates (**Table 1**). At the low P supply, *nirS* gene abundance decreased significantly (by 25%-26%) with biochar addition or mycorrhizal inoculation, but abundance of *nirS* gene increased by 19% with biochar addition at P300 compared to the no biochar treatment (**Figure 4A**). The AMF×BC interaction was a significant source of variation for abundance of *nirK* gene at P300 only (**Table 1**). Compared to the control at P300, the *nirK* gene copy number decreased significantly (by 35% to 59%) in the other treatments (**Figure 4B**). In the field experiment, P addition decreased significantly the abundance of *nirS and nirK* genes by 17% and 45% compared to no P addition, respectively (**Table 2**).

The *nosZ* gene abundance was significantly influenced by the AMF×BC interaction at P40 only, whereas at 300 P both main effects significantly influenced *nosZ* genes (**Table 1**). Compared to the control at P40, the *nosZ* gene abundance increased significantly (by 84% to 96%) in the other treatments. at the high P supply, the number of *nosZ* gene copies increased significantly (by 37% and 65% with biochar addition or mycorrhizal inoculation, respectively) (**Figure 4C**).

The *nosZ/(nirK+nirS)* ratio was significantly influenced by the interaction AMF×BC at P40 and P300 (**Table 1**). Compared to the control, the *nosZ/(nirK+nirS)* ratio increased significantly (by 115% to 256% at P40 and 78% to 102% at P300) in the other treatments (**Figure 4D**). The *nosZ/(nirK+nirS)* ratio at high P level was 4.06 to 7.56 folds higher than at low P level (calculated from **Figure 4**).

In the field experiment, the *nosZ* gene and the *nosZ/(nirK+nirS)* ratio were significantly influenced by the BC×P interaction. Compared to the BC0P0 treatment, the *nosZ* gene and *nosZ/(nirK+nirS)* ratio increased significantly by 43% and 226% in the BC+P+ treatment. The *nosZ* gene and nosZ/(nirK+nirS) ratio increased significantly by 35% and 172% with P addition compared to no P addition treatment (**Table 2**).

### 3.7 Factors affecting soil N_2_O emissions

At the low P supply, N_2_O emission was correlated positively with shoot growth, root growth and *nirS* gene abundance. The *nosZ* gene copy number, *nosZ/(nirK+nirS)* ratio, soil NO_3_^–^N, NH_4_^+^-N, SOC and AMF colonization were correlated negatively with N_2_O emission. At the high P supply, the *nosZ* abundance and *nosZ/(nirK+nirS)* ratio were correlated negatively with N_2_O emission, whereas shoot growth, root morphology, *nirK* copy number and soil NO_3_^–^N were correlated positively with N_2_O emission (**Figure S2A**). In the field experiment, N_2_O emission was correlated positively with *nirK* and *nirS* genes, and was correlated negatively with wheat yield, *nosZ* and *nosZ/(nirK+nirS)* ratio (**Figure S2B**).

**Figure 5:**
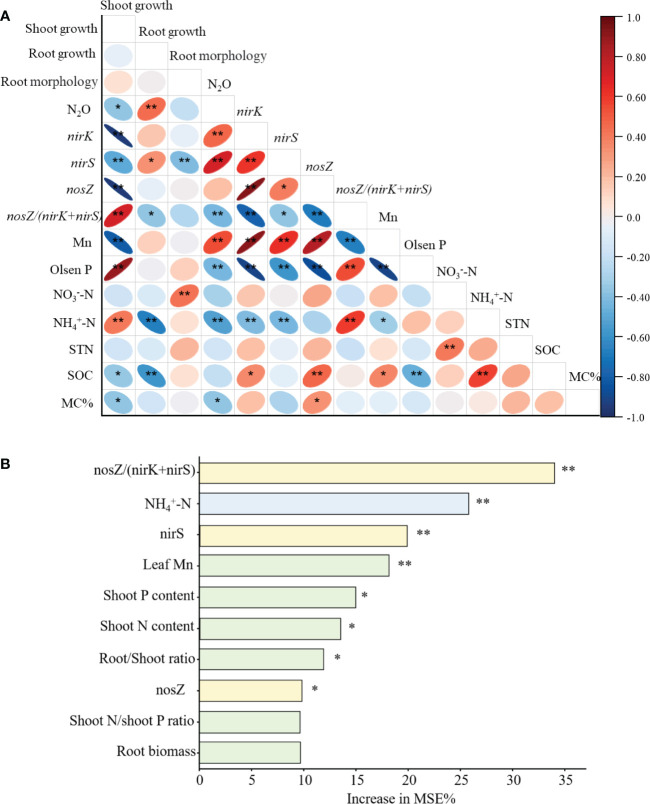
**(A)** Heat map of Pearson’s correlation coefficients with original data among N_2_O emission, gene copies, plant traits and soil properties. **(B)** Random Forest for importance of the specific variables to N_2_O emission. The variables were sorted in the decreasing order of the importance value. **P < 0.01 and *P < 0.05.

When the data from the two P treatments of greenhouse experiment were combined across 25 variables, the abundances of *nirk* and *nirS* genes were correlated positively with N_2_O emission, but *nosZ/(nirk+nirS)* ratio showed a negative correlation with N_2_O emission. Root growth was correlated with N_2_O positively, and shoot growth and AMF colonization were correlated negatively. The mature leaf Mn content was correlated positively with N_2_O. Additionally, Olsen P and soil NH_4_^+^-N showed a negative correlation with N_2_O (**Figure 5A**).

Most important variables to influence soil N_2_O emissions based on the random forest model were ranked in order of importance (**Figure 5B**). The *nosZ/(nirK+nirS)* ratio, soil NH_4_^+^-N, *nirS* abundance and mature leaf Mn concentration were the significant factors associated with N_2_O emission. Shoot P and N content, root/shoot ratio, and *nosZ* copy numbers were the additional significant factors influencing N_2_O emission (**Figure 5B**).

​In summary, the addition of biochar and/or AMF had a negative effect on the total root length for both P40 and P300. The AMF addition decreased leaf Mn concentration, whereas biochar addition increased leaf Mn concentration at P40, suggesting biochar and AMF addition promote P uptake. Compared with P40, the high P significantly promoted wheat growth (16-34%), nutrient content (33-218%) and yield (33-41%), but inhibited soil N_2_O emissions (32-95%). However, biochar and/or AMF addition reduced soil N_2_O emissions by 24-93% and 32%, respectively, in greenhouse and field experiments. This decrease was associated mainly with the diminished abundance of *nirK* and *nirS* (17-59%) and the increased *nosZ* (35-65%) in the greenhouse and field experiments, respectively.

## 4 Discussion

### 4.1 The growth of plants in different environments

In the present study, the results showed that the wheat root and shoot biomass growth and yield were all higher at the high than low P supply regardless of the biochar or AMF additions (**Figure 1**), suggesting the inhibiting effect of low P on plant growth. In general, AMF has a negative effect on plant growth in P-rich soils, whereas it promotes nutrient acquisition in the low-P soils (Johnson et al., 2015). However, in the greenhouse experiment, AMF additions (AMF added alone or in combination with BC) reduced biomass and nutrient content at both low and high P rates (**Figure 1** and **Table 1**). Compared to the control, AMF addition significantly decreased shoot biomass and P content at the low and high P supply (**Figure 1**). AMF inoculation could inhibit the root nutrients absorption in the high nutrient environment, while maintaining the contribution of AMF absorption pathway, finally, this negative effect influenced the crop growth (Smith et al., 2011). The root length decreased significantly by mycorrhizal inoculation in the present study (**Figure 2**), hinting a trade-off between AMF and root length in absorption of available P (Li et al., 2019; Zhang et al., 2023). We also found that leaf Mn concentration was significantly reduced by AMF inoculation as well, especially in low P environment (**Figure 2E**). Leaf Mn concentration has been proposed as a signature for the strength of carboxylates exudation in the rhizosphere (Lambers et al., 2021). It means that mycorrhizal inoculation reduced the rhizosphere carboxylate releasing of wheat root. Similar trend was also found in the previous work on *Kennedia* species that the AMF inoculation reduced exudation of organic carboxylates up to 50% (Ryan et al., 2012). However, the reduction in the release of carboxylate may decrease the ability of plants to utilize insoluble phosphorus (Lambers et al., 2008). As a thin-root species, wheat mainly depends more on the root system in P acquisition compared with thick-root species (Wen et al., 2019). Therefore, AMF inoculation may suppress wheat growth through inhibiting the root growth and exudation, and then bring a negative effect on wheat growth.

In the different P environments, adding biochar alone had no significant effect on shoot N and P content in wheat shoots in the greenhouse (**Figure 1**), but biochar and P addition could promote wheat yield in the field (**Table 2**). Biochar addition could significantly reduce root biomass, total root length (**Figures 2A, B**), which suggested that biochar addition can diminish carbon partitioning to roots. Our findings in previous field experiment also indicated that biochar addition decreased assimilate partitioning to rice roots at elongation stage (Liu et al., 2021). That might have been attributed to biochar addition improving root activity (Cao et al., 2019). For instance, our results showed that biochar increasing leaf Mn concentration (**Figure 2E**). This indicated that the addition of biochar could increase the release of carboxylate. We also found that biochar addition can enhance the AMF colonization (**Figure 2F**), which may be caused by biochar can change soil nutrient availability (such as P available, **Figure S1**) and be a refuge for colonizing fungi (Warnock et al., 2007).

Meanwhile, our results showed a negative relationship between root tissue density and root diameter in both low and high P environments (**Table S2**). This may be because the increase in root diameter was mainly driven by the high thickness or proportion of root cortex (Kong et al., 2019). Due to the cortex density was significantly lower than stele density in root structure, suggesting that an increase of cortex thickness meant a decrease in root tissue density (Kong et al., 2019). Meanwhile, the higher proportion or thickness of cortex can provide more niche space for AMF colonization and facilitate higher mycorrhizal colonization intensity (Kong et al., 2017). In the present study, AMF colonization was the highest, while RTD decreased under AMF and biochar combined treatment at the low P supply (**Figures 2B, F**), reflecting a integrated variation of root morphology and AMF in plant P-acquisition strategy.

### 4.2 N_2_O emission and driving factors

In both greenhouse and field experiments, N_2_O emission flux was much higher at the low P supply compared to the high P supply (**Figure 3**), which was consistent with the results reported elsewhere (Shen et al., 2021; Shen and Zhu, 2022). The suppression of soil N_2_O emissions by P addition can partly be attributed to the improved shoot N content and thus decreased availability of N substrates (Xiao et al., 2022a). In the present study, the shoot N content and yield at the high P supply was indeed significantly higher at high compared with low P supply (**Figure 1**), providing a support for the above viewpoint.

In addition, compared to low P supply, the abundance of *nirK* and *nirS* genes associated with N_2_O production decreased by 17% to 59%, but the *nosZ* genes associated with N_2_O consumption (N_2_O→N_2_) in denitrification were increased by 34% to 1340% at the high P supply, which result in the *nosZ/(nirK+nirS)* ratio at the high P supply was 2.72 to 7.56 times greater than that at the low P supply (**Figure 4** and **Table 2**), suggesting a strong influence of P addition on N cycling genes involved in denitrification (Xiao et al., 2022b). In the present study, among the top five factors affecting the N_2_O emission, *nosZ/(nirK+nirS)*, *nirS*, leaf Mn concentration and stem P content were closely related to soil Olsen P across all the treatments (**Figure 5B**). Therefore, these findings suggest that the variation of soil P environment dominated N_2_O emission by altering proportion of functional microbes in the total microbiome, nutrient acquisition strategy, and plant growth.

As **Figure 3** shown, AMF addition significantly decreased N_2_O emissions, especially at the low P supply. There was no evidence of a positive effect of AMF on crop growth in the present study (**Figure 1**); therefore, the suppressed effect of AMF on N_2_O emission may be not *via* promoting plant N retention and reducing availability of soil N for N_2_O production. Nevertheless, we found that AMF decreased the copy number of *nirK* genes and increased *nosZ* genes, thus reducing N_2_O emission by regulating the denitrification process (**Figure 4**). Zhao et al. (2021) and Storer et al. (2018) both reported that AMF could directly influence denitrification and reduce N_2_O emission, which was consistent with the results in this study.

Biochar application had no significant positive effect on the shoot N and P content regardless of the P supply in greenhouse experiment (**Table 1**). However, a decrease in N_2_O emission by biochar addition was likely associated with a decreased copy number of *nirS* genes at the low P supply and *nirK* genes at the high P supply, and with the increased abundance of the *nosZ* gene to modify the denitrification process (**Figure 4**). For instance, N_2_O emission had a negative correlation with *nosZ* gene and positive correlation with *nirK* genes in the field experiment (**Figure S2B**), which was consistent with our previous research in the field (Liu et al., 2020). In addition, biochar application could significantly reduce P availability in soil, especially in low P environment, which might bring about P deficiency stress, and then reduced activity of denitrifying microorganisms led to decreased soil N_2_O emissions (Wang et al., 2022).

Because biochar enhanced AMF colonization (**Figure 2F**), AMF and biochar showed a significant interaction on N_2_O emission at the low P supply (**Table 1**). The inhibition effect of AMF plus biochar application on N_2_O emission was stronger than that of biochar application alone (**Figure 3A**). This may be attributed mainly to a decreased copy number of *nirS* genes in the either AMF or BC treatment. In summary, the effect of AMF and biochar on suppressing N_2_O emission was mainly *via* regulating denitrification process rather than reducing substrates for denitrification by promoting plant growth.

## 5 Conclusion

The eight-week greenhouse and one-year field experiments verified that the high P supply suppressed soil N_2_O emissions *via* promoting plant growth, root nutrient acquisition capacity and yield, and also through regulating denitrification process, especially increasing the *nosZ/(nirK+nirS)* ratio compared to the low P supply. The soil N_2_O emissions was mitigate after AMF and/or biochar addition regardless of P supply in greenhouse or in field experiments, but the effect of under high P supply which might be attributed mainly to the decreased copy numbers of genes associated with N_2_O production *(nirK* and *nirS*) and the increased copy number of the *nosZ* gene associated with N_2_O consumption in the process of denitrification. Our findings highlight that strong interaction among plant, soil microbiome and soil properties in regulating denitrification.

## Data availability statement

The original contributions presented in the study are included in the article/**Supplementary Material**. Further inquiries can be directed to the corresponding author.

## Author contributions

ZH: conceptualization, writing-original draft, review & editing. ZD: data analysis. SH: data analysis. AZ: conceptualization and writing-review & editing. All authors contributed to the article and approved the submitted version.

## Supplementary material

Supplementary Table 1 Primers used in qPCR. Note: R=A or G; S=C or G.

Supplementary Table 2 Pearson’s correlation with original data among N_2_O emission, genes, plant traits and soil properties under 40P treatment (upper-right part) and 300P treatment (lower-left part). Note: GR: nosZ/(nirK+nirS). SB, shoot biomass; RB, root biomass; TRL, total root length; RD, root diameter; RV, root volume; SRL, specific root length; RTD, root tissue density; R/S, root/shoot ratio; P con, Phosphorus concentration; P acc, Phosphorus accumulation; N con, nitrogen concentration; N acc, nitrogen accumulation; N/P, shoot N/shoot P ratio; C%, shoot C content; Mn, leaf Mn concentrations; OP, Olsen phosphorus; STN, shoot total nitrogen content; SOC, soil organic carbon; MC, mycorrhizal colonization.

Supplementary Figure 1 Variation in the soil NO_3_^–^N and NH_4_^+^-N, Olsen P, soil organic carbon, and root diameter as influenced by P rates (40 and 300 mg kg^−1^ soil) and AMF and BC additions in the greenhouse experiment. Each value is the mean of five replicates ( ± SE). ** *P*<0.01 and * *P*<0.05.

Supplementary Figure 2
**(A)** Heat map of Pearson’s correlation coefficients among N_2_O emission, gene copies, plant traits and soil properties under P300 treatment (upper-right part) and P40 treatment (lower-left part) under greenhouse experiment. Each value is the mean of five replicates( ± SE) **(B)** Heat map of Pearson’s correlation coefficients with original data among yield, N_2_O emission, gene copies in one-year field experiment. Each value is the mean of four replicates ( ± SE). Note: shoot growth as a principal component includes shoot biomass, shoot phosphorus concentration and content, and shoot nitrogen concentration and content. Root growth as a principal component includes root biomass, total root length and root/shoot ratio. Root morphology as a principal component includes root diameter, specific root length and root tissue density. Mn, mature leaf Mn concentrations; STN, soil total nitrogen content; SOC, soil organic carbon; MC%, mycorrhizal colonization. ** *P*<0.01 and * *P*<0.05.

## References

[B1] Abalos D. van Groenigen J. W. Philippot L. Lubbers I. M. De Deyn G. B. (2019). Plant trait-based approaches to improve nitrogen cycling in agroecosystems. J. Appl. Ecol. 56 (11), 2454–2466. doi: 10.1111/1365-2664.13489

[B2] An N. Zhang L. Liu Y. Shen S. Li N. Wu Z. C.. (2022). Biochar application with reduced chemical fertilizers improves soil pore structure and rice productivity. Chemosphere 298, 134304. doi: 10.1016/j.chemosphere.2022.134304 35301997

[B3] Auge R. M. (2004). Arbuscular mycorrhizae and soil/plant water relations. Can. J. Soil Sci. 84 (4), 373–381. doi: 10.4141/S04-002

[B4] Bender S. F. Plantenga F. Neftel A. Jocher M. Oberholzer H. R. Kohl L.. (2014). Symbiotic relationships between soil fungi and plants reduce N2O emissions from soil. Isme J. 8 (6), 1336–1345. doi: 10.1038/ismej.2013.224 24351937 PMC4030222

[B5] Cao H. Ning L. Xun M. Feng F. Li P. Yue S.. (2019). Biochar can increase nitrogen use efficiency of malus hupehensis by modulating nitrate reduction of soil and root. Appl. Soil Ecol. 135, 25–32. doi: 10.1016/j.apsoil.2018.11.002

[B6] Case S. D. C. McNamara N. P. Reay D. S. Stott A. W. Grant H. K. Whitaker J. (2015). Biochar suppresses N_2_O emissions while maintaining n availability in a sandy loam soil. Soil Biol. Biochem. 81, 178–185. doi: 10.1016/j.soilbio.2014.11.012

[B7] Chen H. Zhang W. Gurmesa G. A. Zhu X. Li D. Mo J. (2017). Phosphorus addition affects soil nitrogen dynamics in a nitrogen-saturated and two nitrogen-limited forests. Eur. J. Soil Sci. 68 (4), 472–479. doi: 10.1111/ejss.12428

[B8] Coskun D. Britto D. T. Shi W. Kronzucker H. J. (2017). How plant root exudates shape the nitrogen cycle. Trends Plant Sci. 22, 661–673. doi: 10.1016/j.tplants.2017.05.004 28601419

[B9] Dai L. Li H. Tan F. Zhu N. He M. Hu G. (2016). Biochar: a potential route for recycling of phosphorus in agricultural residues. GCB Bioenergy 8, 852–858. doi: 10.1111/gcbb.12365

[B10] Davidson E. A. (2009). The contribution of manure and fertilizer nitrogen to atmospheric nitrous oxide since 1860. Nat. Geosci. 2, 659–662. doi: 10.1038/ngeo608

[B11] Dong Z. Li H. Xiao J. Sun J. Liu R. Zhang A. (2022). Soil multifunctionality of paddy field is explained by soil pH rather than microbial diversity after 8-years of repeated applications of biochar and nitrogen fertilizer. Sci. Total Environ. 853, 158620. doi: 10.1016/j.scitotenv.2022.158620 36084779

[B12] Efthymiou A. Jensen B. Jakobsen I. (2018). The roles of mycorrhiza and penicillium inoculants in phosphorus uptake by biochar-amended wheat. Soil Biol. Biochem. 127, 168–177. doi: 10.1016/j.soilbio.2018.09.027

[B13] Gui H. Gao Y. Wang Z. Shi L. Yan K. Xu J. (2021). Arbuscular mycorrhizal fungi potentially regulate N_2_O emissions from agricultural soils *via* altered expression of denitrification genes. Sci. Total Environ. 774, 145133. doi: 10.1016/j.scitotenv.2021.145133 33610977

[B14] Jiang Z. Lian F. Wang Z. Xing B. (2020). The role of biochars in sustainable crop production and soil resiliency. J. Exp. Bot. 71, 520–542. doi: 10.1093/jxb/erz301 31232450

[B15] Johnson N. C. Wilson G. W. Wilson J. A. Miller R. M. Bowker M. A. (2015). Mycorrhizal phenotypes and the l aw of the m inimum. New Phytol. 205, 1473–1484. doi: 10.1111/nph.13172 25417818

[B16] Kong D. Wang J. Wu H. Valverde-Barrantes O. J. Wang R. Zeng H.. (2019). Nonlinearity of root trait relationships and the root economics spectrum. Nat. Commun. 10, 2203. doi: 10.1038/s41467-019-10245-6 31101818 PMC6525182

[B17] Kong D. Wang J. Zeng H. Liu M. Miao Y. Wu H.. (2017). The nutrient absorption–transportation hypothesis: optimizing structural traits in absorptive roots. New Phytol. 213, 1569–1572. doi: 10.1111/nph.14344 27859373

[B18] Kool D. M. Dolfing J. Wrage N. Van Groenigen J. W. (2011). Nitrifier denitrification as a distinct and significant source of nitrous oxide from soil. Soil Biol. Biochem. 43, 174–178. doi: 10.1016/j.soilbio.2010.09.030

[B19] Kormanik P. P. McGraw A. (1982). Quantification of vesicular-arbuscular mycorrhizae in plant roots. In: Schenck NC (ed) Methods and Principles of Mycorrhiza Research. American Phytopathological Society, St Paul, pp 37–45.

[B20] Lambers H. Hayes P. E. Laliberte E. Oliveira R. S. Turner B. L. (2015). Leaf manganese accumulation and phosphorus-acquisition efficiency. Trends Plant Sci. 20, 83–90. doi: 10.1016/j.tplants.2014.10.007 25466977

[B21] Lambers H. Raven J. A. Shaver G. R. Smith S. E. (2008). Plant nutrient-acquisition strategies change with soil age. Trends Ecol. Evol. 23, 95–103. doi: 10.1016/j.tree.2007.10.008 18191280

[B22] Lambers H. Shane M. W. Cramer M. D. Pearse S. J. Veneklaas E. J. (2006). Root structure and functioning for efficient acquisition of phosphorus: matching morphological and physiological traits. Ann. Bot. 98, 693–713. doi: 10.1093/aob/mcl114 16769731 PMC2806175

[B23] Lambers H. Wright I. J. Guilherme Pereira C. Bellingham P. J. Bentley L. P. Boonman A.. (2021). Leaf manganese concentrations as a tool to assess belowground plant functioning in phosphorus-impoverished environments. Plant Soil 461, 43–61. doi: 10.1007/s11104-020-04690-2

[B24] Lehmann J. Joseph S. (2009). “Biochar for environmental management: an introduction,” in Biochar for environmental management. science and technology. Eds. Lehmann J. Joseph S. (London: Earthscan), pp 1–pp12.

[B25] Lehmann J. Rillig M. C. Thies J. Masiello C. A. Hockaday W. C. Crowley D. (2011). Biochar effects on soil biota–a review. Soil Biol. Biochem. 43, 1812–1836. doi: 10.1016/j.soilbio.2011.04.022

[B26] Li H. Ma Q. Li H. Zhang F. Rengel Z. Shen J. (2013). Root morphological responses to localized nutrient supply differ among crop species with contrasting root traits. Plant Soil 376, 151–163. doi: 10.1007/s11104-013-1965-9

[B27] Liu L. Gundersen P. Zhang T. Mo J. (2012). Effects of phosphorus addition on soil microbial biomass and community composition in three forest types in tropical China. Soil Biol. Biochem. 44, 31–38. doi: 10.1016/j.soilbio.2011.08.017

[B28] Liu B. Li H. Li H. Zhang A. Rengel Z. (2021). Long-term biochar application promotes rice productivity by regulating root dynamic development and reducing nitrogen leaching. GCB Bioenergy 13, 257–268. doi: 10.1111/gcbb.12766

[B29] Liu H. Li H. Zhang A. Rahaman M. A. Yang Z. (2020). Inhibited effect of biochar application on N_2_O emissions is amount and time-dependent by regulating denitrification in a wheat-maize rotation system in north China. Sci. Total Environ. 721, 137636. doi: 10.1016/j.scitotenv.2020.137636 32172102

[B30] Li H. Zhang D. Wang X. Li H. Rengel Z. Shen J. (2019). Competition between *Zea mays* genotypes with different root morphological and physiological traits is dependent on phosphorus forms and supply patterns. Plant Soil 434, 125–137. doi: 10.1007/s11104-018-3616-7

[B31] Martínez-García L. B. De Deyn G. B. Pugnaire F. I. Kothamasi D. van der Heijden M. G. (2017). Symbiotic soil fungi enhance ecosystem resilience to climate change. Global Change Biol. 23, 5228–5236. doi: 10.1111/gcb.13785 PMC5697572 28614605

[B32] Morris E. K. Morris D. J. P. Vogt S. Gleber S. C. Bigalke M. Wilcke W.. (2019). Visualizing the dynamics of soil aggregation as affected by arbuscular mycorrhizal fungi. ISME J. 13, 1639–1646. doi: 10.1038/s41396-019-0369-0 30742058 PMC6775962

[B33] Okiobe S. T. Pirhofer-Walzl K. Leifheit E. Rillig M. C. Veresoglou S. D. (2022). Proximal and distal mechanisms through which arbuscular mycorrhizal associations alter terrestrial denitrification. Plant Soil. 476, 315–336. doi: 10.1007/s11104-022-05534-x

[B34] Philibert A. Loyce C. Makowski. D. (2013). Prediction of N_2_O emission from local information with random forest. Environ. pollut. 177, 156–163. doi: 10.1016/j.envpol.2013.02.019 23500053

[B35] Ryan M. H. Graham J. H. (2018). Little evidence that farmers should consider abundance or diversity of arbuscular mycorrhizal fungi when managing crops. New Phytol. 220, 1092–1107. doi: 10.1111/nph.15308 29987890

[B36] Ryan M. H. Tibbett M. Edmonds-tibbett T. Suriyagoda L. D. B. Lambers H. Cawthray G. R.. (2012). Carbon trading for phosphorus gain: the balance between rhizosphere carboxylates and arbuscular mycorrhizal symbiosis in plant phosphorus acquisition. Plant Cell Environ. 35, 2170–2180. doi: 10.1111/j.1365-3040.2012.02547.x 22632405

[B37] Senbayram M. Chen R. Budai A. Bakken L. Dittert K. (2012). N_2_O emission and the N_2_O/(N_2_O+N_2_) product ratio of denitrification as controlled by available carbon substrates and nitrate concentrations. Agricult. Ecosyst. Environ. 147, 4–12. doi: 10.1016/j.agee.2011.06.022

[B38] Shen Y. Xu T. Chen B. Zhu B. (2021). Soil N_2_O emissions are more sensitive to phosphorus addition and plant presence than to nitrogen addition and arbuscular mycorrhizal fungal inoculation. Rhizosphere 19, 100414. doi: 10.1016/j.rhisph.2021.100414

[B39] Shen J. Yuan L. Zhang J. Li H. Bai Z. Chen X.. (2011). Phosphorus dynamics: From soil to plant. Plant Physiol. 156, 997–1005. doi: 10.1104/pp.111.175232 21571668 PMC3135930

[B40] Shen Y. Zhu B. (2021). Arbuscular mycorrhizal fungi reduce soil nitrous oxide emission. Geoderma 402, 115179. doi: 10.1016/j.geoderma.2021.115179

[B41] Shen Y. Zhu B. (2022). Effects of nitrogen and phosphorus enrichment on soil N_2_O emission from natural ecosystems: A global meta-analysis. Environ. pollut. 301, 118993. doi: 10.1016/j.envpol.2022.118993 35183669

[B42] Shi Y. Liu X. Zhang Q. (2019). Effects of combined biochar and organic fertilizer on nitrous oxide fluxes and the related nitrifier and denitrifier communities in a saline-alkali soil. Sci. Total Environ. 686, 199–211. doi: 10.1016/j.scitotenv.2019.05.394 31176819

[B43] Smith S. E. Jakobsen I. Grønlund M. Smith F. A. (2011). Roles of arbuscular mycorrhizas in plant phosphorus nutrition: interactions between pathways of phosphorus uptake in arbuscular mycorrhizal roots have important implications for understanding and manipulating plant phosphorus acquisition. Plant Physiol. 156, 1050–1057. doi: 10.1104/pp.111.174581 21467213 PMC3135927

[B44] Smith S. E. Read D. J. (2008). Mycorrhizal symbiosis. Ed 3 (New York: Academic Press).

[B45] Storer K. Coggan A. Ineson P. Hodge A. (2018). Arbuscular mycorrhizal fungi reduce nitrous oxide emissions from N_2_O hotspots. New Phytol. 220, 1285–1295. doi: 10.1111/nph.14931 29206293 PMC6282961

[B46] Sun J. Li H. Wang Y. Du Z. Rengel Z. Zhang A. (2022). Biochar and nitrogen fertilizer promote rice yield by altering soil enzyme activity and microbial community structure. GCB Bioenergy 14, 1266–1280. doi: 10.1111/gcbb.12995

[B47] Ullah B. Shaaban M. Hu R. Zhao J. Lin S. (2016). Assessing soil nitrous oxide emission as affected by phosphorus and nitrogen addition under two moisture levels. J. Integr. Agric. 15, 2865–2872. doi: 10.1016/S2095-3119(16)61353-9

[B48] Wang R. Bicharanloo B. Hou E. Jiang Y. Dijkstra F. A. (2022). Phosphorus supply increases nitrogen transformation rates and retention in soil: A global meta-analysis. Earth's Future 10, e2021EF002479. doi: 10.1029/2021EF002479

[B49] Wang X. X. Li H. Chu Q. Feng G. Kuyper T. W. Rengel Z. (2020). Mycorrhizal impacts on root trait plasticity of six maize varieties along a phosphorus supply gradient. Plant Soil 448, 71–86. doi: 10.1007/s11104-019-04396-0

[B50] Wang X. X. Zhang J. Wang H. Rengel Z. Li H. (2021). Plasticity and co-variation of root traits govern differential phosphorus acquisition among 20 wheat genotypes. Oikos. doi: 10.1111/oik.08606

[B51] Warnock D. D. Lehmann J. Kuyper T. W. Rillig M. C. (2007). Mycorrhizal responses to biochar in soil–concepts and mechanisms. Plant Soil 300, 9–20. doi: 10.1007/s11104-007-9391-5

[B52] Wen Z. Li H. Shen Q. Tang X. Xiong C. Li H.. (2019). Tradeoffs among root morphology, exudation and mycorrhizal symbioses for phosphorus-acquisition strategies of 16 crop species. New Phytol. 223, 882–895. doi: 10.1111/nph.15833 30932187

[B53] Xiao J. Dong S. Shen H. Li S. Wessell K. Liu S.. (2022b). N addition overwhelmed the effects of p addition on the soil c, n, and p cycling genes in alpine meadow of the qinghai-Tibetan plateau. Front. Plant Sci. 13, 860590. doi: 10.3389/fpls.2022.860590 35557731 PMC9087854

[B54] Xiao J. Dong S. Shen H. Li S. Zhi Y. Mu Z.. (2022a). Phosphorus addition promotes nitrogen retention in alpine grassland plants while increasing n deposition. Catena 210, 105887. doi: 10.1016/j.catena.2021.105887

[B55] Xuan Y. Mai Y. Xu Y. Zheng J. He Z. Shu L.. (2022). Enhanced microbial nitrification-denitrification processes in a subtropical metropolitan river network. Water Res. 222, 118857. doi: 10.1016/j.watres.2022.118857 35868099

[B56] Zhang H. Chen C. Gray E. M. Boyd S. E. Yang H. Zhang D. (2016). Roles of biochar in improving phosphorus availability in soils: A phosphate adsorbent and a source of available phosphorus. Geoderma 276, 1–6. doi: 10.1016/j.geoderma.2016.04.020

[B57] Zhang L. Chu Q. Zhou J. Rengel Z. Feng G. (2021). Soil phosphorus availability determines the preference for direct or mycorrhizal phosphorus uptake pathway in maize. Geoderma 403, 115261. doi: 10.1016/j.geoderma.2021.115261

[B58] Zhang A. Wang X. Zhang. D. Dong Z. Ji H. Li H. (2023). Localized nutrient supply promotes maize growth and nutrient acquisition by shaping root morphology and physiology and mycorrhizal symbiosis. Soil Tillage Res. 225, 105550. doi: 10.1016/j.still.2022.105550

[B59] Zhao R. T. Li X. Bei S. K. Li D. D. Li H. G. Christie P.. (2021). Enrichment of nosZ-type denitrifiers by arbuscular mycorrhizal fungi mitigates N_2_O emissions from soybean stubbles. Environ. Microbiol. 23 (11), 6587–6602. doi: 10.1111/1462-2920.15815 34672071

