# Supplementary material for: Effects of biochar and arbuscular mycorrhizal fungi on winter wheat growth and soil N2O emissions in different phosphorus environments
Source: Front Plant Sci. 2022 Dec 14;13:1069627. doi: 10.3389/fpls.2022.1069627 (PMC9795251; doi:10.3389/fpls.2022.1069627)
Supplement: Supplementary Table 1 — Primers used in qPCR. Note: R=A or G; S=C or G. [file DataSheet_1.docx]

**Supporting material**

**Table S1** Primers used in qPCR

| Gene | Name | Sequence | Reference |
| --- | --- | --- | --- |
| *nirK* | nirKF1aCu | 5'-ATC ATG GTS CTG CCG CG-3' | Hallin and Lindgren, 1999 |
|  | nirKR3Cu | 5'-GCC TCG ATC AGR TTG TGG TT-3' |  |
| *nirS* | nirSCd3Af | 5'-GTS AAC GTS AAG GAS ACS GC-3' | Guo et al., 2011 |
|  | nirSR3cd | 5'-GAS TTC GGR TGS GTC TTG A-3' |  |
| *nosZ* | nosZ-F | 5'-AGA ACG ACC AGC TGA TCG ACA-3' | Kloos et al., 2001 |
|  | nosZ-R | 5'-TCC ATG GTG ACG CCG TGG TTC-3' |  |

Note: R=A or G; S=C or G.

**Fig. S1** Variation in the soil NO_3_^-^-N and NH_4_^+^-N, Olsen P, soil organic carbon, and root diameter as influenced by P rates (40 and 300 mg kg^−1^ soil) and AMF and BC additions in the greenhouse experiment. Each value is the mean of five replicates (±SE). ** *P*<0.01 and * *P*<0.05.

**Fig. S2** (a) Heat map of Pearson’s correlation coefficients among N_2_O emission, gene copies, plant traits and soil properties under P300 treatment (upper-right part) and P40 treatment (lower-left part) under greenhouse experiment. Each value is the mean of five replicates(±SE) (b) Heat map of Pearson’s correlation coefficients with original data among yield, N_2_O emission, gene copies in one-year field experiment. Each value is the mean of four replicates (±SE).


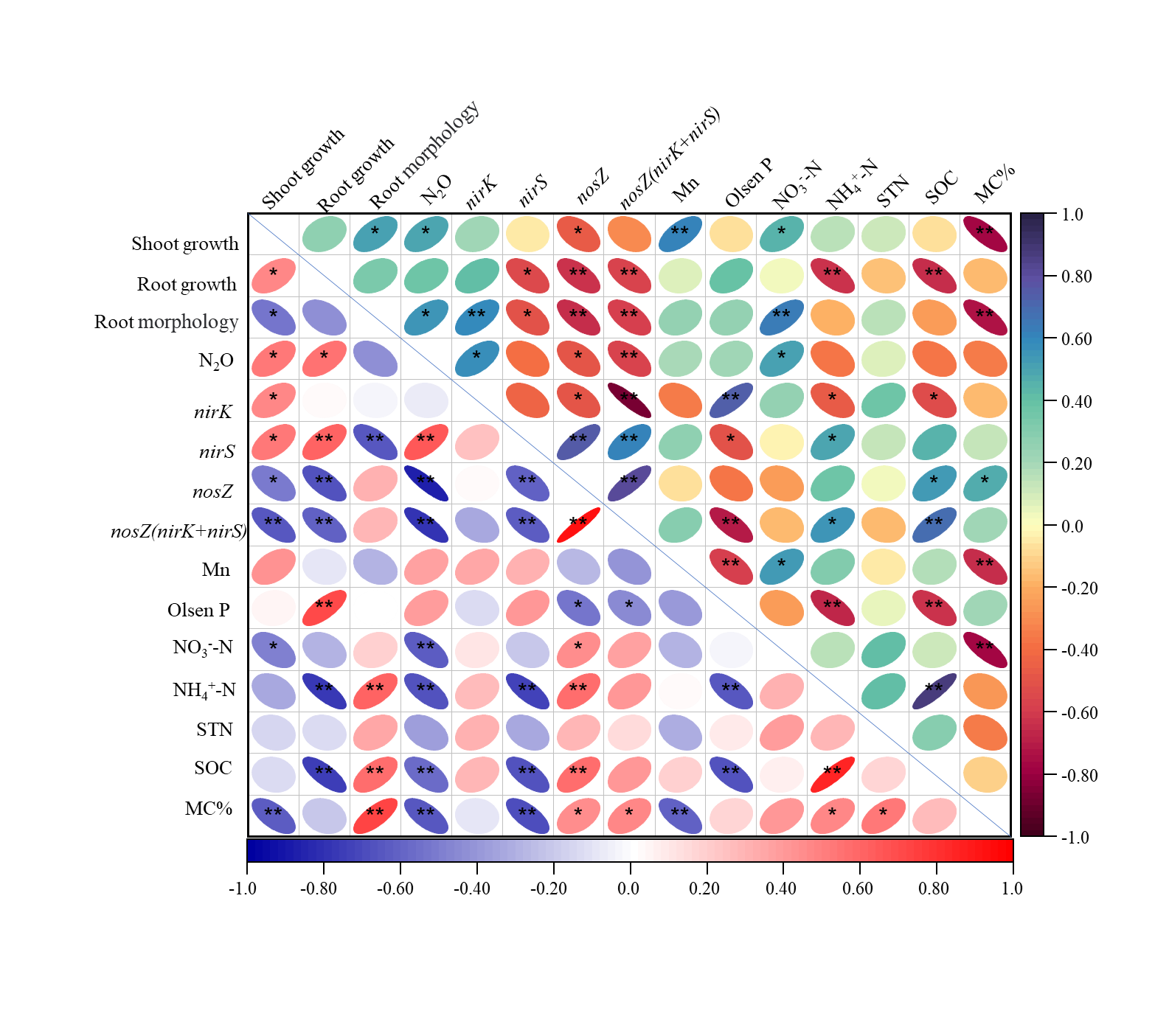


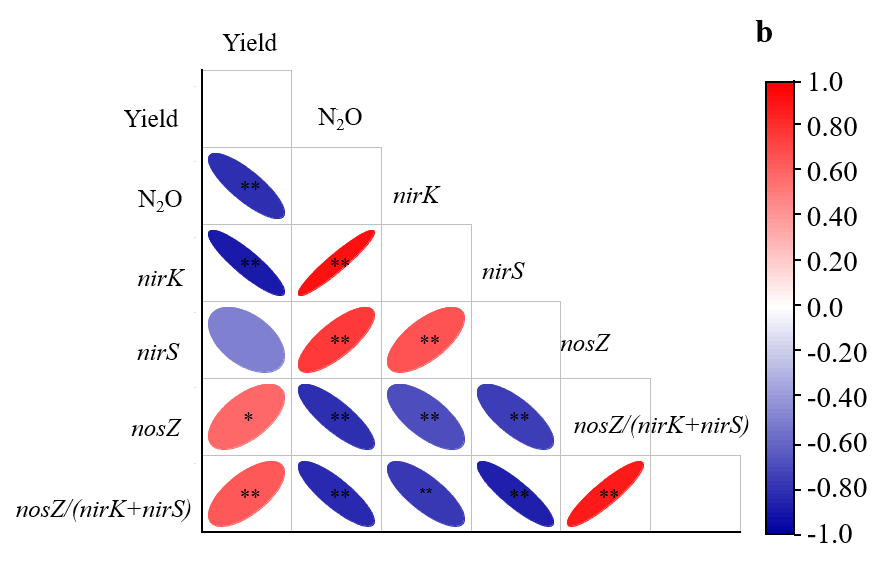


Note: shoot growth as a principal component includes shoot biomass, shoot phosphorus concentration and content, and shoot nitrogen concentration and content. Root growth as a principal component includes root biomass, total root length and root/shoot ratio. Root morphology as a principal component includes root diameter, specific root length and root tissue density. Mn: mature leaf Mn concentrations. STN: soil total nitrogen content. SOC: soil organic carbon. MC%: mycorrhizal colonization. ** *P*<0.01 and * *P*<0.05.

**Table S2.** Pearson’s correlation with original data among N_2_O emission, genes, plant traits and soil properties under 40P treatment (upper-right part) and 300P treatment (lower-left part)


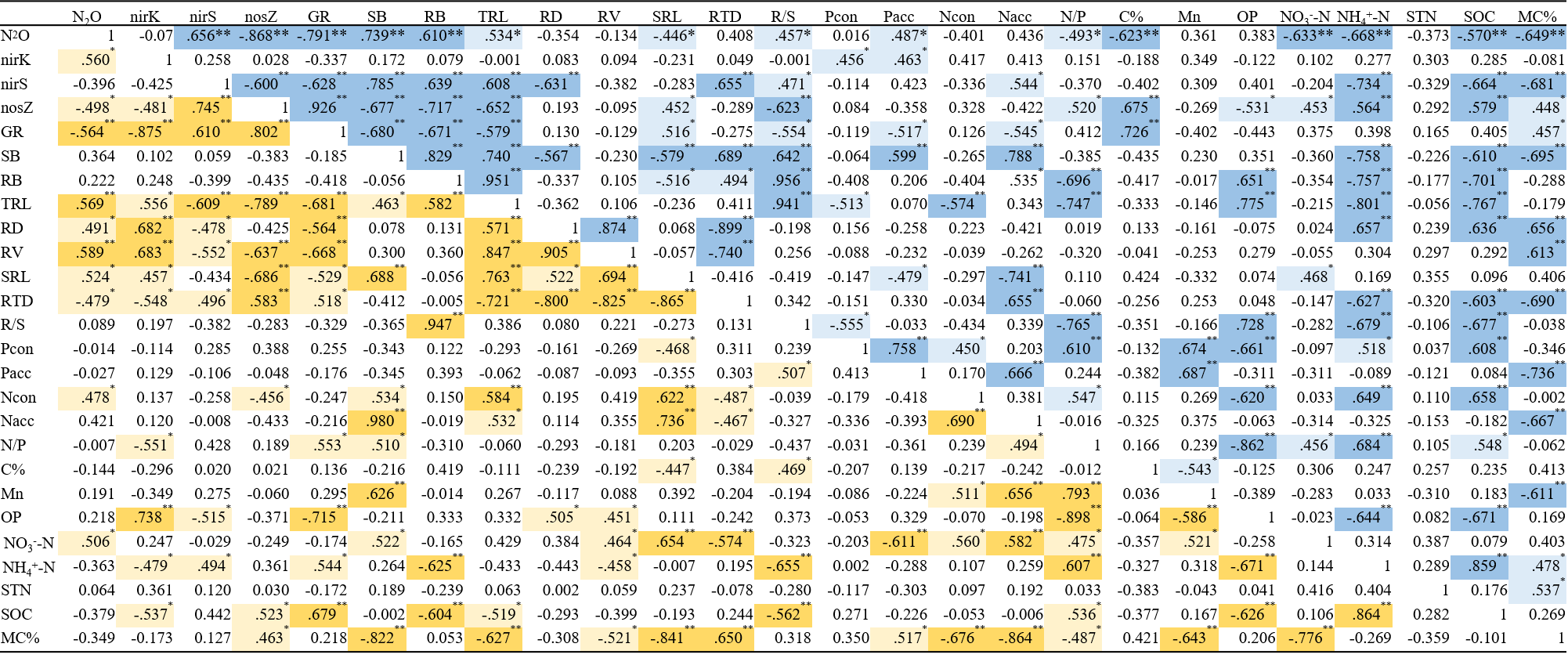


Note: GR: nosZ/(nirK+nirS). SB: shoot biomass. RB: root biomass. TRL: total root length. RD: root diameter. RV: root volume. SRL: specific root length. RTD: root tissue density. R/S: root/shoot ratio. P con: Phosphorus concentration. P acc: Phosphorus accumulation. N con: nitrogen concentration. N acc: nitrogen accumulation. N/P: shoot N/ shoot P ratio. C%: shoot C content. Mn: leaf Mn concentrations. OP: Olsen phosphorus. STN: shoot total nitrogen content. SOC: soil organic carbon. MC: mycorrhizal colonization.
